# Outcomes of esophagectomy after chemotherapy with biweekly docetaxel plus cisplatin and fluorouracil for advanced esophageal cancer: a retrospective cohort analysis

**DOI:** 10.1186/s12957-018-1420-8

**Published:** 2018-07-02

**Authors:** Yuji Akiyama, Akira Sasaki, Fumitaka Endo, Haruka Nikai, Satoshi Amano, Akira Umemura, Shigeaki Baba, Takehiro Chiba, Toshimoto Kimura, Takeshi Takahara, Hiroyuki Nitta, Koki Otsuka, Masaru Mizuno, Yusuke Kimura, Keisuke Koeda, Takeshi Iwaya

**Affiliations:** 1 Department of Surgery, Iwate Medical University School of Medicine, 19-1 Uchimaru, Morioka, Iwate 020-8505 Japan; 2 Department of Palliative Medicine, Iwate Medical University School of Medicine, Morioka, Iwate Japan; 3 Department of Medical Safety Science, Iwate Medical University School of Medicine, Morioka, Iwate Japan

**Keywords:** Biweekly DCF, Preoperative chemotherapy, Esophageal cancer, Esophagectomy

## Abstract

**Background:**

Docetaxel, cisplatin, and 5-fluorouracil (DCF) therapy can cause severe adverse events, including neutropenia and febrile neutropenia. The feasibility of DCF therapy is a concern, particularly for elderly patients, patients with moderate organ disorders, and patients suffering from malnutrition caused by dysphagia or insufficient oral intake. We introduced a biweekly DCF therapy (bDCF) for the purpose of reducing severe adverse events for these fragile patients. This study investigated the feasibility and outcome of an esophagectomy after bDCF therapy for patients with advanced esophageal squamous cell carcinoma.

**Methods:**

Fifty-nine patients with esophageal carcinoma underwent an esophagectomy after DCF or bDCF therapy as primary chemotherapy. DCF was administered to 37 patients in the DCF group, whereas bDCF was administered to 22 patients in the bDCF group.

**Results:**

Patients in the bDCF group were significantly older than those in the DCF group (*p* = 0.016). Heart and pulmonary comorbidities were significantly more common in the bDCF than in the DCF group (*p* < 0.001 and *p* = 0.039, respectively). Grade 3 or 4 neutropenia was less frequent in the bDCF than in the DCF group (40.9 vs. 81.1%, *p* = 0.002). Anorexia was more frequent in the DCF group than in the bDCF group (18.9 vs. 0%, *p* = 0.030). The clinical response rate of the bDCF group was significantly higher than that of the DCF group (86.4 vs. 62.2%, *p* = 0.047). There was no significant between-group difference in the postoperative morbidity rate (bDCF 45.5% vs. DCF 32.4%) or in the histological therapeutic effect.

**Conclusion:**

The results demonstrate that primary bDCF therapy for high-risk patients with advanced esophageal cancer is feasible and safe in both chemotherapeutic and perioperative periods without a reduction in the efficacy of DCF therapy.

## Background

Recent studies have reported that combination chemotherapy using docetaxel/cisplatin/5-fluorouracil (DCF) is effective as preoperative chemotherapy for advanced esophageal cancer [1–4]. Hara et al. [2] evaluated the feasibility of preoperative chemotherapy with DCF for esophageal squamous cell carcinoma (ESCC) and reported estimated 2-year progression-free survival and overall survival (OS) rates of 74.5 and 88%, respectively. Primary DCF therapy for patients with unresectable esophageal cancer resulted in conversion surgery for 41.7% (20/48) of patients, in which R0 resection was achieved in 19 patients (39.6%) [5]. Based on these results, the use of preoperative DCF therapy for advanced ESCC has been increasing. However, DCF therapy can cause severe adverse events, such as grade 3 or 4 neutropenia (range 66.6–78.2%) and febrile neutropenia (FN) (range 14.5–22.9%) [1, 5].

Elderly patients and patients with comorbidities account for a large proportion of patients with esophageal cancer [6, 7]. An increase in DCF-induced adverse events is anticipated in elderly patients, patients with organ disorders, and patients suffering from malnutrition. Myelosuppression in DCF therapy is mainly caused by docetaxel. Several studies have reported that a modified regimen in which docetaxel was divided and administered biweekly reduced DCF toxicity [8–12]. Hironaka et al. [10] reported a reduced incidence (25.5%) of grades 3 and 4 neutropenia and no cases of FN in a phase I/II trial of biweekly DCF (bDCF) regimen for metastatic esophageal cancer without a decrease in antitumor activity of the standard DCF therapy.

Recent studies have reported a relatively high incidence (approximately 30–50%) of infectious complications after an esophagectomy for esophageal cancer [13–16]. It has been reported that postoperative infectious complications were not only associated with postoperative mortality but also overall long-term survival [13, 17]. Furthermore, patients with postoperative infections who received preoperative chemotherapy had a poorer overall survival [18]. Immunosuppression induced by a preoperative chemotherapeutic treatment can increase postoperative infections. Therefore, myelosuppression and gastrointestinal side effects caused by preoperative DCF therapy may worsen postoperative infections leading to life-threatening diseases in high-risk patients with esophageal cancer and may reduce long-term survival benefits by increasing the incidence of postoperative infections. With the aim of reducing severe adverse events, we introduced bDCF therapy for high-risk patients (e.g., elderly patients, patients with organ disorders, and patients suffering from malnutrition). Here, we investigated the feasibility and safety of esophagectomy after bDCF for patients with advanced ESCC.

## Methods

### Patients

**Fig. 1:**
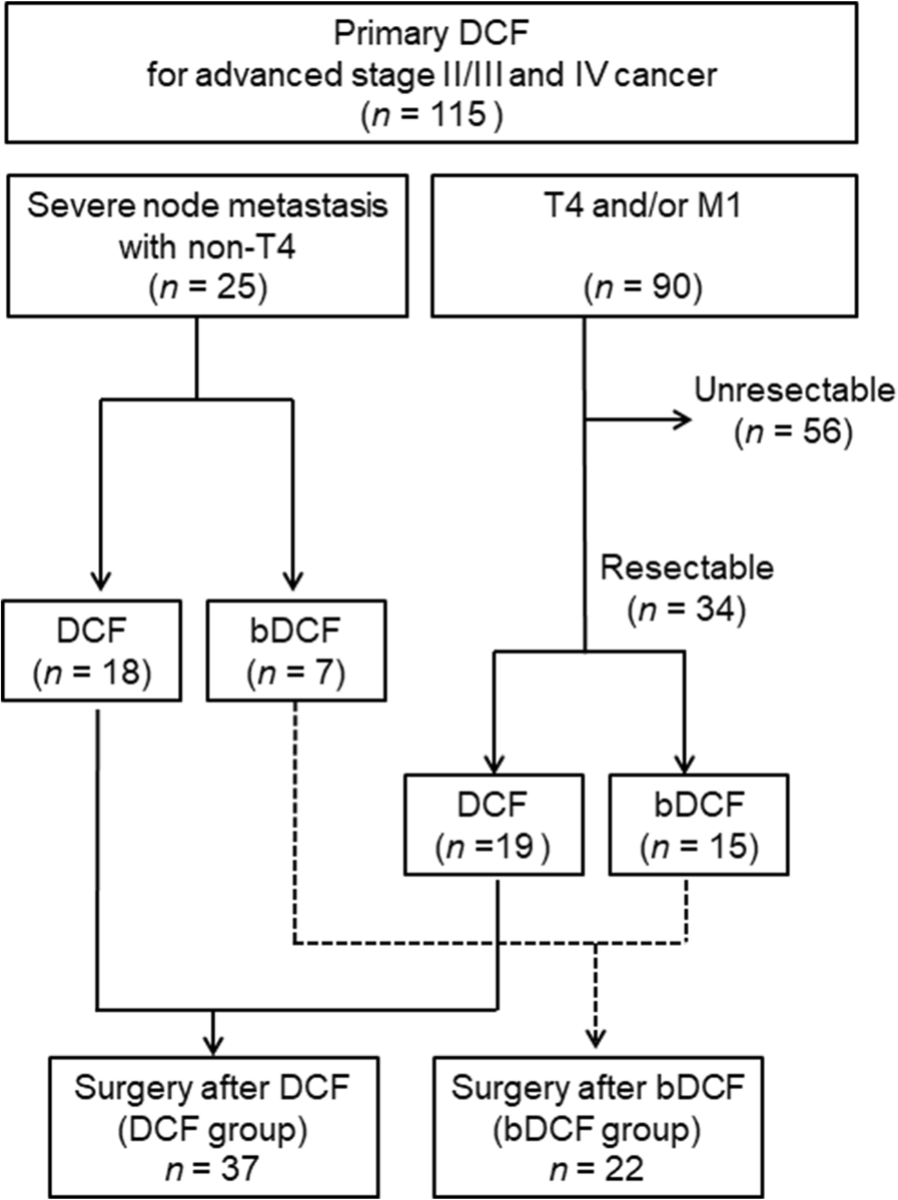
Flow diagram of primary docetaxel, cisplatin, and 5-fluorouracil therapy. DCF, chemotherapy with docetaxel, cisplatin, and 5-fluorouracil; bDCF, biweekly DCF

We retrospectively reviewed 115 consecutive patients with ESCC who received DCF or bDCF therapy as primary chemotherapy at the Department of Surgery, Iwate Medical University Hospital, between March 2007 and October 2017. In total, 59 patients who underwent esophagectomy after receiving chemotherapy were analyzed by dividing into DCF (DCF group, *n* = 37) and bDCF regimens (bDCF group, *n* = 22) (Fig. 1). The remaining 56 patients did not undergo esophagectomy because they did not exhibit adequate respiratory or cardiac functions for surgery, did not consent to undergo surgery, or showed persistent T4 cancer and/or distant metastasis despite receiving DCF or bDCF. These patients were sequentially treated with other chemotherapy, chemoradiotherapy, or the best supportive care. The clinical characteristics of the patients are presented in Table 1. The tumor location was classified according to the Japanese Classification of Esophageal Cancer (JCEC), 11th edition [19]. Clinical stages were classified according to the Union for International Cancer Control classification, 7th edition.

**Table 1:** Clinical characteristics of the patients included in the study

	DCF, *N* = 37	bDCF, *N* = 22	*p* value
Age (years)^a^	61.2 ± 9.5	67.1 ± 7.3	0.016
Sex			0.725
Male/female	30/7	17/5	
Tumor location^b^			0.045
Ut/Mt./Lt/Ae	5/21/10/1	2/10/4/6	
Clinical T stage^c^			0.502
T1/T2/T3/T4a/T4b	1/3/14/8/11	1/0/6/5/10	
Clinical N stage^c^			0.625
N0/N1/N2/N3	2/29/5/1	2/15/5/0	
Clinical M stage^c^			0.599
M0/M1 (LYM)	34/4	21/1	
Clinical stage^c^			0.151
IIA/IIB/IIIA/IIIB/IIIC/IV	1/3/20/2/8/3	1/0/7/1/12/1	
ASAPS			0.082
1/2/3	13/21/3	2/18/2	
Comorbidities			
Heart disease	8	15	< 0.001
Pulmonary disease	1	4	0.039
Diabetes mellitus	5	5	0.362

*Ae* abdominal esophagus, *ASAPS* American Society of Anesthesiologists performance states, *Lt* lower thoracic esophagus, *LYM* lymph node metastasis, *Mt*. middle thoracic esophagus, *Ut* upper thoracic esophagus

^a^Mean ± standard deviation

^b^According to the Japanese Classification of Esophageal Cancer, 11th edition

^c^According to the UICC classification, 7th edition

### Chemotherapy

The criteria for selection of DCF or bDCF for patients with advanced ESCC have been described previously [20]. In brief, patients treated with DCF or bDCF as the primary chemotherapy regimen presented with at least one of the following lesions: T4 (or suspected T4) tumor; the involvement of more than three metastatic lymph nodes; metastatic lymph nodes over two fields of the mediastinal, abdominal, and cervical regions; or bulky metastatic nodes of > 3 cm in diameter or with extranodal invasion. Meanwhile, patients with stage II and stage III non-T4 cancer and with fewer than two metastatic lymph nodes (< 2 cm in diameter and limited to a single region) were treated with cisplatin and 5-fluorouracil (CF) regimen [21]. The patients with stage I cancer who were treated with surgery alone without preoperative chemotherapy and those with stage II or III cancer who were treated with CF were excluded from this study. In the DCF group, the regimen consisted of intravenous docetaxel (60–70 mg/m^2^) on day 1, intravenous cisplatin (80 mg/m^2^) on day 1, and continuous infusion of 5-fluorouracil (800 mg/m^2^) on days 1–5. The regimen was repeated every 3–4 weeks. The criteria for the selection of the bDCF regimen were as follows: elderly patients older than 75 years; patients with a respiratory functional disorder or severe pulmonary emphysema, as shown by computed tomography (CT); patients with a heart disease comorbidity such as myocardial infarction or angina pectoris; patients with a cerebral infarction comorbidity prescribed anticoagulant or antiplatelet treatment; and patients with dysphagia or insufficient oral intake due to esophageal stenosis caused by cancer. In the bDCF group, the regimen consisted of docetaxel (30 mg/m^2^) on days 1 and 15 in combination with CF (80 mg/m^2^ cisplatin on day 1 and 800 mg/m^2^ 5-fluorouracil on days 1–5) repeated every 4 weeks [10]. Granulocyte colony-stimulating factor (G-CSF) was administered in case of grade 4 neutropenia or FN. Prophylactic ciprofloxacin was administered on days 5–15. 5-FU, cisplatin, and docetaxel doses were reduced by 20% in the next course if grade 3 FN, grade 4 neutropenia, anemia, or thrombocytopenia was observed. Docetaxel and 5-fluorouracil doses were reduced by 20% if grade 3 or 4 mucositis oral or diarrhea was observed. The cisplatin dose was reduced by 20% if creatinine clearance (Ccr) was 50 ≤ Ccr < 60, by 40% if Ccr was 40 ≤ Ccr < 50, and terminated if Ccr was < 40 mL/min. Patients with esophageal stenosis due to cancer were administered an enteral diet via a nasal feeding tube until they could manage an oral diet. All patients underwent CT before the next course to evaluate the clinical response; this was done following the Response Evaluation Criteria in Solid Tumors v. 1.1. Adverse events were assessed by the Common Terminology Criteria for Adverse Events v. 4.0. Histological therapeutic effects were defined according to the JCEC, 11th edition [19, 22]. In a histological study, squamous cell carcinomas were graded as well, moderately, or poorly differentiated according to the amount of keratin present [22]. Lymphatic vessel invasion and blood vessel invasion were classified according to the guidelines for clinical and pathological studies on carcinomas of the esophagus in Japan [22].

### Surgical procedure

In this study, esophagectomy was performed by two surgeons in the same group. A radical esophagectomy via a right thoracotomy or thoracoscopy was performed following the Guidelines for Diagnosis and Treatment of Carcinoma of the Esophagus in Japan [23]. A thoracoscopic esophagectomy was performed in the left decubitus position until 2009 and in the prone position after 2010, as previously reported [24–26]. The reconstruction conduit was a gastric tube pulled through the posterior mediastinum or via the retrosternal route with cervical esophagogastrostomy [27]. A left thoracoabdominal approach or transhiatal resection was performed for patients with cancer at the esophagogastric junction. In the left thoracoabdominal approach using the thoracolaparotomy procedure, a lower esophagectomy and total gastrectomy with a lower mediastinal and abdominal lymphadenectomy and Roux–en–Y jejunal reconstruction were performed. The perioperative management during radical esophagectomy has been described previously [28]. Postoperative complications were defined based on the classification of the Esophagectomy Complications Consensus Group [29]. *Clostridium difficile* enteritis was defined as the presence of enteritis by laboratory detection of the *C*. *difficile*-positive toxin in the stool or a *C*. *difficile* positive stool culture. Infectious complication grades were defined following the Clavien–Dindo classification [30].

### Statistical analysis

Statistical analyses were performed using the SAS statistical analysis software, JMP 10 (SAS, Cary, NC, USA). Differences in patient characteristics and outcomes between the two groups were estimated using the *χ*^2^ test, Student’s *t* test, or Wilcoxon’s rank test. *p* < 0.05 was considered statistically significant.

## Results

### Patient characteristics

The patients in the bDCF group were significantly older than those in the DCF group (*p* = 0.016, Table 1). Tumors were more frequently observed in the abdominal esophagus of patients in the bDCF than in the DCF group. There was no significant difference in clinical stage between the two treatment groups. There were 19 patients and 15 patients with T4 cancer in the DCF and bDCF groups, respectively. Although a significant difference was not observed in the American Society of Anesthesiologists performance status between the two groups, it was generally higher in the bDCF group than in the DCF group. Heart and pulmonary comorbidities were significantly more common in the bDCF than in the DCF group (*p* < 0.001 and *p* = 0.039, respectively).

### Adverse events of chemotherapy

Adverse events related to chemotherapy are listed in Table 2. No significant differences were found between the groups in the number of courses, discontinuation of the next course due to adverse events, or dose reduction rate. Although grade 3 or 4 neutropenia was less frequently observed in the bDCF group compared with that in the DCF group (40.9 vs. 81.1%, *p* = 0.002), there was no significant difference in FN frequency (9.1 vs. 13.5%, Table 2). G-CSF was administered significantly more frequently in the DCF group (60/87 courses, 69%) as compared with the bDCF group (13/59 courses, 22%, *p* < 0.001, Table 3). The mean period of G-CSF use was also longer in the DCF group than in the bDCF group (4.5 days vs. 2.8 days, *p* = 0.001, Table 3). There was no significant difference in the administration of antibiotics except for a prophylactic treatment between both groups. Among non-hematologic toxicities, grade 3 or 4 anorexia was more frequent in the DCF than in the bDCF group (18.9 vs. 0%, *p* = 0.030, Table 2). There was no significant between-group difference in preoperative white blood cell counts or neutrophil counts. However, the period between day 1 of the last course of chemotherapy and the day of operation tended to be shorter in the bDCF group (bDCF 45.1 days vs. DCF 52.3 days; Table 2).

**Table 2:** Adverse events associated with chemotherapy

	DCF, *N* = 37	bDCF, *N* = 22	*p* value
Number of courses^a^	2.3 ± 1.3	2.7 ± 1.2	0.303
Discontinuance of next course due to AE	6 (16.2)	2 (9.1)	0.440
Dose reduction	8 (21.6)	3 (13.6)	0.446
Hematologic toxicity (grades 3–4)			
Neutropenia	30 (81.1)	9 (40.9)	0.002
Febrile neutropenia	5 (13.5)	2 (9.1)	0.612
Anemia	1 (2.7)	0	0.437
Thrombocytopenia	1 (2.7)	0	0.437
Non-hematologic toxicity (grades 3–4)			
Nausea/vomiting	1 (2.7)	1 (4.5)	0.705
Diarrhea	10 (27)	5 (22.7)	0.714
Mucositis oral	6 (16.2)	1 (4.5)	0.180
Anorexia	7 (18.9)	0	0.030
Days to operation^ab^ (range)	52.3 ± 14.7 (30–86)	45.1 ± 13.5 (30–83)	0.067
Preoperative white blood cell count (/μL)^a^	5929.2 ± 2094	5017.3 ± 1777.7	0.091
Preoperative neutrophil count (/μL)^a^	3529.9 ± 1819.5	2950.2 ± 1313.1	0.199

*AE* adverse events

^a^Mean ± standard deviation

^b^Time period between day 1 at last course of chemotherapy and operation

**Table 3:** Administration of granulocyte colony-stimulating factor (G-CSF) and antibiotics

	DCF (%)	bDCF (%)	*p* value
Total number of courses	87	59	
Administration of G-CSF			< 0.001
(+)	60 (69)	13 (22)	
(−)	27 (31)	46 (78)	
Period of administration (days)^a^	4.5 ± 2.8	2.8 ± 1.2	0.001
Administration of antibiotics^b^			0.373
(+)	15 (17.2)	7 (11.9)	
(−)	72 (82.8)	52 (88.1)	
Period of administration (days)^a^	6 ± 3.3	6.6 ± 2.4	0.683
Hospital stay in each course (days)^a^ (range)	18.4 ± 6.2 (9–50)	16.3 ± 5.5 (9–32)	0.051

^a^Mean ± standard deviation

^b^Administration of antibiotics except for prophylactic treatment

### Chemotherapy efficacy

The efficacy of chemotherapy is indicated in Table 4. The clinical response rate of the bDCF group was significantly higher than that of the DCF group (86.4 vs. 62.2%, *p* = 0.047). There was no significant between-group difference in the histological therapeutic effects. Pathological complete response (grade 3) was achieved in 22.7% of patients in the bDCF group and 13.5% in the DCF group. There were no significant differences in the differentiation grade, lymphatic vessel invasion, blood vessel invasion, or pathological stage between the groups (Table 4).

**Table 4:** Efficacy of chemotherapy and pathological findings

	DCF (%), *N* = 37	bDCF (%), *N* = 22	*p* value
Clinical response			
CR/PR/SD/PD	2/21/13/1	0/19/3/0	
Response rate			0.047
CR + PR	23 (62.2)	19 (86.4)	
Histological therapeutic effect^a^			0.312
Grade 0/1a/1b/2/3	3/16/6/7/5	1/4/6/6/5	
Grade of differentiation^a^			0.593
Well differentiated	6	5	
Moderately differentiated	17	7	
Poorly differentiated	8	4	
Not determined	6	6	
Lymphatic vessel invasion^a^			0.358
ly0/ly1/ly2/ly3	16/16/2/3	13/7/2/0	
Blood vessel invasion^a^			0.117
v0/v1/v2/v3	16/20/1/0	12/7/3/0	
pT stage^b^			0.542
T0/Tis/T1/T2/T3/T4a	5/0/6/8/17/1	5/1/5/3/8/0	
pN stage^b^			0.219
N0/N1/N2/N3	17/16/3/1	11/8/0/3	
pStage^b^			0.606
0/IA/IB/IIA/IIB/IIIA/IIIB/IIIC/IV	5/5/3/5/4/11/1/2/1	4/2/2/3/5/2/0/2/2	

*CR* complete response, *PD* progression disease, *PR* partial response, *SD* stable disease

^a^According to the Japanese Classification of Esophageal Cancer, 11th edition

^b^According to the UICC classification, 7th edition

### Surgical and postoperative outcomes

The surgical procedures and postoperative outcomes are presented in Table 5. All patients underwent complete resection. None of the groups experienced intraoperative morbidity. Morbidity rate in the DCF group was 32.4% and that in the bDCF group was 45.5%; one patient in the bDCF group presented with postoperative pneumonia and vocal cord palsy (Table 5). There was no significant difference in the postoperative morbidity rate between the groups (*p* = 0.317). There was also no significant between-group difference in the overall rate of infectious complications, including pneumonia, wound infections, and *C*. *difficile* enteritis (bDCF 31.8 vs. DCF 13.5%, Table 6). *C*. *difficile* enteritis occurred more often in the bDCF group (three patients, 13.6%), and all the patients exhibited grade II Clavien–Dindo classification (Table 6). In all cases, the infections were resolved following oral administration of vancomycin. Operative mortality was zero in both groups (Table 5). The postoperative hospital stay was significantly shorter for the bDCF group (19.4 days; range 10–33 days) as compared with that for the DCF group (28.4 days; range 12–106 days; *p* = 0.008, Table 5).

**Table 5:** Surgical procedures and postoperative outcomes

	DCF (%), *N* = 37	bDCF (%), *N* = 22	*p* value
Surgical procedure			0.224
Right thoracotomy	6 (16.2)	1 (4.5)	
Thoracoscopy	29 (78.4)	17 (77.3)	
Left thoracotomy	2 (5.4)	3 (13.6)	
Transhiatal resection	0	1 (4.5)	
Morbidity	12 (32.4)	10 (45.5)	0.317
Pneumonia	5 (13.5)	2 (9.1)	0.612
Dysrhythmia atrial	1 (2.7)	0	0.437
Anastomotic leak	0	0	–
Chyle leak	1 (2.7)	0	0.437
Vocal cord palsy	5 (13.5)	4 (18.2)	0.630
Bleeding requiring reoperation	0	0	–
Wound infection	0	2 (9.1)	0.062
*C*. *difficile* enteritis	0	3 (13.6)	0.021
Operative mortality	0	0	–
Postoperative hospital stay (days^a^, range)	28.4 ± 18.4 (12–106)	19.4 ± 18.4 (10–33)	0.008

*C*. *difficile*, *Clostridium difficile*

^a^Mean ± standard deviation

**Table 6:** Postoperative infectious complications

	DCF (%), *N* = 37	bDCF (%), *N* = 22	*p* value
Overall infectious disease	5 (13.5)	7 (31.8)	0.091
Pneumonia	5 (13.5)	2 (9.1)	0.612
Wound infection	0	2 (9.1)	0.062
*C*. *difficile* enteritis	0	3 (13.6)	0.021
Clavien–Dindo classification			0.217
Grade I	0	0	
Grade II	4	7	
Grade IIIb	1	0	

*C*. *difficile*, *Clostridium difficile*

## Discussion

According to the results of clinical trials of adjuvant chemotherapy for patients with resectable stage II/III thoracic esophageal cancer by the Japan Clinical Oncology Group (JCOG), adjuvant chemotherapy with CF improved the disease-free survival rate of patients compared with surgery alone (JCOG9204) and preoperative CF resulted in a higher overall survival rate compared with postoperative CF (JCOG9907) [21, 31]. In the JCOG9907 study, the survival benefits of neoadjuvant CF were observed only in patients with stage II cancer. These reports have suggested that a more powerful preoperative treatment was required for patients with stage III esophageal carcinoma. Therefore, in recent years, DCF has been used as the primary chemotherapy regimen for patients with greater than stage III advanced esophageal carcinoma. DCF therapy has a reportedly high clinical efficacy, with a total response rate of 53.7–64.3% in advanced esophageal cancer [1, 2]. Although DCF therapy also showed a high incidence of treatment-related severe toxicity, particularly myelosuppression, DCF has been widely used as an esophageal cancer treatment, and side effects can be reduced by the use of supporting therapy, such as G-CSF, antiemetic agents, antibiotics, and nutritional supplements [2, 5, 32]. Aspiration pneumonia, enteritis, and infection via the catheter are common in the treatment course of patients with esophageal cancer. Several high-risk patients, such as elderly patients, patients with serious comorbidities, and patients with malnutrition caused by esophageal stenosis, have received esophageal cancer treatment in recent clinical practices. Myelosuppression caused by DCF therapy may increase the severity of infections in such patients, thereby leading to life-threatening diseases towing to the fragile status of these patients. Even in patients without infections, prolonged myelosuppression may delay the initiation of scheduled therapy, including chemotherapy, radiation, and surgery. Several studies have demonstrated a dramatic decrease in the incidence of neutropenia and FN with bDCF therapy (docetaxel administered dividedly) as compared with standard DCF therapy [8–12]. Thus, bDCF can be safely used even for high-risk patients with advanced esophageal cancer.

In the present study, grade 3 or 4 neutropenia was less frequent in the bDCF than in the DCF group (40.9 vs. 81.1%, *p* = 0.002, Table 2). Although the chemotherapeutic agent (i.e., docetaxel) was also administered on day 15 in the bDCF group, the period between day 1 of the last course of chemotherapy and the day of surgery was shorter for the bDCF rather than the DCF group (Table 2). Anorexia was also less frequent in the bDCF than in the DCF group. Previous research reported that nutritional support for the patients with decreased oral intake during chemotherapy could reduce hematological toxicities, including neutropenia, although the mechanism of action of nutritional support remains unclear [33]. The present study also demonstrated that the frequency and period of G-CSF administration were reduced in the bDCF group (Table 3). Reduced anorexia by bDCF therapy might be favorable to oral intake and nutritional status of the patients. The administration of docetaxel in separate doses may reduce myelosuppression by maintaining nutritional status by reducing anorexia. In terms of an antitumor effect, the clinical response rate in the present study was higher in the bDCF than in the DCF group. Furthermore, there was no significant between-group difference in the histological therapeutic effect (Table 4). These findings suggest that bDCF can reduce the adverse effects frequently observed with DCF therapy without reducing the chemotherapeutic antitumor effect of DCF.

In the present study, we also focused on differences in perioperative outcomes between the DCF and bDCF groups. Although there were more high-risk patients in the bDCF than in the DCF group, there was no increase in postoperative morbidity in the bDCF group. From the point of view of postoperative infections, the overall severity of infectious diseases was Clavien–Dindo grade II in the bDCF group (Table 6). *C*. *difficile* enteritis was only observed in the bDCF group (Table 6). Dineen et al. [34] reported that the frequency of *C*. *difficile* infection had increased in the last decade and that the mortality rate associated with *C*. *difficile* enteritis was high. The reported risk factors for *C*. *difficile* infection were receiving chemotherapy, acid reduction by proton pump inhibitors or H2-blockers, previous use of antibiotics, and gastrointestinal/abdominal surgery [34–37]. In the present study, patients with postoperative *C*. *difficile* infection exhibited all the aforementioned risk factors. Two of three (66.7%) patients with postoperative *C*. *difficile* enteritis infection also had *C*. *difficile* enteritis during bDCF chemotherapy. Previous research reported that rates of recurrent *C*. *difficile* infection were 10–20% and that the risk factors for recurrence were older age and illness severity [38]. Our results indicated that gastrointestinal side effects of preoperative bDCF therapy might increase postoperative *C*. *difficile* enteritis after an esophagectomy. Because patients with more advanced cancer and higher risk were included in the bDCF group, it was predicted that the prolongation of hospital stay and increase of postoperative morbidity would be more frequently observed in the bDCF group. However, such a deterioration of outcomes was not observed in the bDCF group. Longer hospital stay was observed in the DCF group than in the bDCF group (Table 5); this might be caused by severe vocal cord palsy in the DCF group.

The usefulness of modified DCF therapy in which docetaxel was administered in divided doses has been previously reported in patients with lung adenocarcinoma and gastroesophageal adenocarcinoma [8, 9, 39, 40]. The bDCF regimen may be a promising and effective treatment for reducing toxicities without decreasing therapeutic effect regardless of the target organ and histological types of cancer.

## Conclusion

The findings of the present study suggest that primary bDCF therapy for high-risk patients with advanced ESCC is feasible and safe in both chemotherapeutic and perioperative periods, without a reduction of the efficacy of the DCF therapy.

## Abbreviations

bDCF: Biweekly docetaxel, cisplatin, and 5-fluorouracil
Ccr: Creatinine clearance
CT: Computed tomography
DCF: Chemotherapy with docetaxel, cisplatin, and 5-fluorouracil
ESCC: Esophageal squamous cell carcinoma
FN: Febrile neutropenia
G-CSF: Granulocyte colony-stimulating factor
JCEC: Japanese Classification of Esophageal Cancer
OS: Overall survival
